# Optimization of Compression and Flexural Properties of Masonry Veneers with Recycled PET-1

**DOI:** 10.3390/polym15051122

**Published:** 2023-02-23

**Authors:** Juan Paredes, Willan Castillo, Gabriela Salinas, Henry Erazo, Víctor H. Guerrero

**Affiliations:** 1 Escuela Internacional de Doctorado (EIDUNED), Universidad Nacional de Educación a Distancia (UNED), 28040 Madrid, Spain; 2 Facultad de Ingeniería Civil y Mecánica, Universidad Técnica de Ambato, Ambato 180104, Ecuador; 3 GI3M—Grupo de Investigación e Innovación en Ingeniería Mecánica, Universidad Técnica de Ambato, Ambato 180104, Ecuador; 4 Independent Researcher, Ambato 180103, Ecuador; 5 Department of Material, Escuela Politécnica Nacional, Quito 170525, Ecuador

**Keywords:** recycling and reuse of PET-1 materials, masonry veneers, compression strength, flexural strength, experimental design DOE/RSM, composite materials

## Abstract

The study of new materials formulated using recycled polymers offers an ecological and sustainable alternative for the construction industry. In this work, we optimized the mechanical behavior of manufactured masonry veneers made from concrete reinforced with recycled polyethylene terephthalate (PET) from discarded plastic bottles. For this purpose, we used the response surface methodology to evaluate the compression and flexural properties. PET percentage, PET size and aggregate size were used as input factors in a Box–Behnken experimental design resulting in a total of 90 tests. The fraction of the commonly used aggregates replaced by PET particles was 15%, 20% and 25%. The nominal size of the PET particles used was 6, 8 and 14 mm, while the size of the aggregates was 3, 8 and 11 mm. The function of desirability was used to optimize response factorials. The globally optimized formulation contained 15% of 14 mm PET particles in the mixture, and 7.36 mm aggregates, obtaining important mechanical properties of this characterization of masonry veneers. The flexural strength (four-point) was 1.48 MPa, and the compression strength was 3.96 MPa; these values show property improvements of 110% and 94%, respectively, compared to commercial masonry veneers. Overall, this offers the construction industry a robust and environmentally friendly alternative.

## 1. Introduction

Increased consumption of polyethylene terephthalate (PET) bottles is observed on a global scale and is directly related to a continuously increasing amount of plastic garbage, which threatens water and soil. Commodity plastics are not biodegradable; burning them is a nonviable solution as it liberates dangerous gases that are harmful to the air [1,2]. Different plastics have been used as aggregates, fillers or fibers to mix with cement, mortar and concrete after mechanical treatment [3]. Previous authors have investigated these plastics’ use in the construction industry to improve concrete performance [1,4,5]. Incorporating plastic can improve concrete properties because it has high toughness, good behavior for abrasion, low thermal conductivity and high heat capacity [3,6]. It also improves the elastic modulus and resistance to shear and flexural strength [3]. When sand is replaced by crushed PET during concrete beam production, it is not necessary to go higher than 15% to use these materials for structural applications [4].

In this work, we analyzed a polymer (PET) and a ceramic (concrete) to improve the properties of a composite material used in the construction industry. Concrete is easily obtained, has good compression strength, long useful life and a low cost, which is why it is frequently chosen for various applications and is used every day for construction purposes [4,7]. Around ten trillion tons of concrete are produced annually worldwide [8]. However, concrete generates very significant emissions of carbon dioxide (CO_2_), corresponding to 7% of total global emissions [9,10]. On the other hand, PET is a polymer whose main characteristics include a high grade of toughness and excellent resistance to fatigue and tearing. PET has good physical properties in a humid environment and in the presence of acids, fats, oils and solvents [7,11]. The principal use of PET is for plastic bottles to store liquids. However, since the 1970s, when the production and commercialization of PET started, it is believed that the cost of collecting and recycling PET has amounted to 12.7% and 54.5% of the cost of manufacturing plastic bottles using new material, respectively. In other words, the cost of making recycled plastic bottles is 32.8% lower than the cost of the initial product [12]. Besides recycling the polymer for bottles, the use of PET residues has also been investigated in a wide variety of applications. For instance, waste PET bottles were recycled and used to fabricate porous membranes for the filtration of high-temperature solvents [13], to obtain thin-film composite membranes with high chemical and heat resistance [14] and to develop nanofibrous membranes for oil removal [15]. In the same vein, waste PET fabric has been used to fabricate flexible wearable membranes for personal thermal management applications [16]. Since PET has a lower density than sand, research about replacing the sand used in concrete with PET has focused on a substitution based on volume, not weight [7]. The replacement of 5 wt. % of sand with PET implies that the same grain is supported, the resistance is reduced, and fluency and consistency values remain very close to reference values [17]. During reinforced concrete beam production, replacing sand with PET in proportions of up to 15% results in a reduction of 12–21% in compression strength [4]. When PET fibers are used to reinforce mortars, the volumes usually used are 0.5%, 1.0% and 1.5%, which results in a significant increase in flexural strength. The maximum volume should not exceed 1.5 to obtain better workability [18]. Adding fibers from PET bottles to concrete allows us to solve structural problems, such as cracks. The volume percentage of fibers added to concrete directly influences the compression resistance as haulage of concrete, and the fiber length directly influences the tensile strength of concrete [19].

Utilizing experimental, statistical and technological hardware, it is possible to know the properties of a material and to decide if it will be useful in each application [20]. The statistical methodology to optimize experimentation is known as the Design of Experiments (DOE) [21]. DOE is a methodology consisting of performing a series of experiments with binding elements to realize changes debated in the process variables, which is feasible to identify the causes of the changes in the responses [22,23]. DOE is based on experimentation, making it necessary to obtain replies and to randomize information. Across the replies, we can estimate the experimental error. As the reply number increases, the experimental error decreases if the experiments are performed in the same conditions [24]. Ribeiro et al. [25] used Minitab 18 to analyze information, perform a DOE, and analysis of variance (ANOVA) when evaluating reinforcing particles’ effect. The quartz flow analysis demonstrated that DOE was efficient in multivariable problem evaluation since it provides a large amount of information from a small number of tests [26]. The response surface methodology was applied to determine the K factor of the native pozzolan masonry and the durability of concrete. The ideal replacement values were found, obtaining a value of K = 0.2 and managing to optimize replacement levels and treatment time [27]. The Box–Behnken methodology is used to optimize six variables of entry. Optimization implies studying the response of the designed statistical combinations, estimating the coefficients, and fitting the best experimental conditions in a mathematical model to predict the exact model’s response and verify the model’s adequacy [28]. Advanced DOE approaches are also being used to optimize materials and material processing. For instance, Hardian et al. [29] presented a methodology that combined DOE and machine learning to realize the sustainable synthesis of a metal–organic framework. This methodology illustrated the use of artificial intelligence to define the synthesis parameters that would result in a sustainably processed material with optimum characteristics. Mangaraj et al. [30] used a Box–Behnken design and a response surface methodology to determine the plasma arc cutting parameters that would result in an optimum cut quality for an AISI 304 steel. Arunkumar et al. [31] performed a Taguchi optimization to define the welding parameters that would result in the highest impact strength of a weld joint.

In this work, it is possible to minimize environmental contamination by reusing the PET found in plastic bottles deposited daily in unsuitable places taking many years to disintegrate due to their chemical properties. To make the PET concrete mix, we proceed to realize the selection of only the body of the bottle, rejecting the lid (Polypropylene [PP]), the label (High-density polyethylene [HDPE]), and the ring of the peak of the bottle (PP) to later realize the process of grinding. Crushed polyethylene-terephthalate is then partially used to replace the thick aggregation to support the curve granulometry of the original material. Next, the Artesil 1 mold [32] is manufactured to pour the composite material inside and finally perform the flexural strength (four-point) and compression at 21 days. This search follows a DOE experiment design to determine miscellany parameters statistically and with the function of desirability (PET %, PET dimension and aggregation dimension) to establish the ideal combination of entry factors for the properties of flexural (four-point) and compression tests [16,19,28].

## 2. Materials and Methods

### 2.1. Description of Masonry Veneers Materials

The materials used in this work were collected in different cities in Ecuador. The nominal size, shape, density and place of origin of the materials used in this work appear in Table 1.

### 2.2. Material Processing and Sample Preparation

PET particles with nominal sizes of 6, 8 and 14 mm were obtained using a Nelmor G1215 M1 grinding machine. The post-consumer plastic bottles used during the process had nominal volumes between 50 and 3000 mL. The aggregates were sieved to obtain three different sizes: 3, 8 and 11 mm. To prepare the concrete, a water/cement (W/C) dosage of 0.74 was used. The actual density of the mixture is 1860 g/cm^3^. The actual density of the mix (*RDM*) was 1.860 g/cm^3^. The optimum density of the mix (*ODM*) was 0.845 g/cm^3^, causing an optimum percentage of voids (OPV) = 54.6%, calculation obtained according to ASTM C 138/C 138M; however, based on the experience of concrete dosage, an OPV = 23% was determined.

(1) $$OPV=\frac{RDM-ODM}{RDM}*100$$

The amount of paste for different settlements is 275.30 dm^3^. The concrete was reinforced using crushed recycled PET to substitute the aggregates in percentages of 15%, 20% and 25% by volume. Table 2 shows the 15 different combinations of materials used to obtain the manufactured masonry veneers studied in this work. The dosage of each material used was determined via the optimal density method. The amounts of concrete and the reinforcing PET and aggregate particles were weighted using a Hotcom DJ6001A electronic scale, with a capacity of 6000 g and a precision of 0.1 g.

The concrete mixtures were poured into a series of molds made using a silicone elastomer whose commercial name is Artesil 1. The temperature during manufacturing was between 23 and 26 °C. The manufactured composite masonry veneers were demolded after 24 h, followed by curing and drying for 21 days. The final dimensions of the composite veneers were 470 mm × 100 mm × 30 mm. A total of 90 manufactured composite masonry veneer samples were obtained. These veneers were used for flexural (four-point) and compression tests according to NTE INEN 2554:2011 and NTE INEN 1 573:2010, respectively. The flexural and compression tests were performed using a Shimadzu-Concreto 2000X universal testing machine. There were 45 observations for the flexural tests (analyzing maximum flexural stress) and 45 for compression tests (analyzing maximum compression strength).

### 2.3. Experimental Design

The goal of the experiments’ design is to analyze the behavior of the input factors (% of PET, PET size aggregate size) and the experimental responses (flexural and compression strength) to find the best dosage for the manufactured masonry veneer. In this work, we also studied how well the model explains the tests’ information by using the global fit of the model, analyzed via the determination coefficient, R^2^. The tests’ individual optimization was performed with the flexural (four-point) and compression tests to carry out the global optimization, using the desirability function for this purpose. The design was performed using the response surface methodology with the Box–Behnken design. This design is convenient because it has three continuous factors, and the major advantage over other methods is its efficiency with respect to the number of combinations. In addition, containing replicates at the center allows the presence of curvature to be detected. As shown in Table 3, this design must use 3 factors, and 15 treatments with 3 replies each, producing 45 results. A total of 90 manufactured masonry veneers were used.

For the variance analysis of the experimental design for the response surface methodology, the following statistical model was defined for the interest of studying linear, interaction and quadratic effects:

(2) $$Y_{ijk}=\mu +\alpha_{i}+\beta_{j}+\gamma_{k}+{\left(\alpha^{2}\right)}_{i}+{\left(\beta^{2}\right)}_{j}+{\left(\gamma^{2}\right)}_{k}+{\left(\alpha \beta \right)}_{ij}+{\left(\alpha \gamma \right)}_{ik}+{\left(\beta \gamma \right)}_{jk}+{\left(\alpha \beta \gamma \right)}_{ijk}+\varepsilon_{ijkl}$$

where $\;\alpha_{i}$ is the changeability due to PET percentage, $\;\beta_{j}$ is the changeability due to PET dimensions,$\;\mathrm{and}\;\gamma_{k}$ is the changeability due to the Aggregates’ dimensions.

### 2.4. Desirability Function Analysis

The desirability function analysis is based on transforming the responses predicted by the model $\hat{y}$ of each mechanical property of the manufactured masonry veneers tested for the different combinations in dimensionless values inside the interval (0, 1) named as desirability individuals $d_{i}$.

Individual desirability when it is desirable to maximize the exit response:

(3) $$d_{i}=\{\begin{array}{c} 0,\\ {\left(\frac{\hat{y}-y_{min}}{y_{máx}-y_{min}}\right)}^{r}\\ 1, \end{array},y_{min}\leq \begin{array}{c} \hat{y}\leq y_{min}\\ \hat{y}\leq y_{máx}\\ \hat{y}\geq y_{máx} \end{array},r\geq 0$$

Individual desirability when it is desirable to minimize exit response:

(4) $$d_{i}=\{\begin{array}{c} 1,\\ {\left(\frac{\hat{y}-y_{máx}}{y_{min}-y_{máx}}\right)}^{r}\\ 0, \end{array},y_{min}\leq \begin{array}{c} \hat{y}\leq y_{min}\\ \hat{y}\leq y_{máx}\\ \hat{y}\geq y_{máx} \end{array},r\geq 0$$

where $y_{máx}$ is the upper specification of the response, $y_{min}$ is the lower specification of the response, and r is a constant to define the form of the desirability function for each response.

After obtaining individual desirability, a value is defined for average desirability, which transforms the problem of optimizing multiple responses into a problem of a single response that can be analyzed objectively. To calculate global desirability (GD), the following formula is used with the individual desires of each response:

(5) GD=(d1w1∗d2w2∗⋯∗diwi)1w

where $w_{i}$ is the relative importance that has every response and w is the sum of the relative importance.

### 2.5. Scanning Electron Microscopy (SEM)

A VEGA 3 SBU TESCAN scanning electron microscope (SEM) was used in a low vacuum condition as it is a non-conductive material, with SE (secondary electron detector) and HV = 5.0 Kv, this in order to analyze the morphology of referential materials, with respect to the conditions and results of the global optimum (GO). These cases analyzed (Table 2) are Case 10, similar to GO in all conditions, and Case 5, not similar to the GO in the conditions, in order to relate the adhesion and cohesion between the matrix and components (PET) of the masonry veneer. The detail of this analysis is specified later in item 3.4.

## 3. Results

Using the response surface methodology (RSM) with the Box–Behnken design, the number of tests for the characterization of flexure and compression of the masonry veneers was determined: 15 cases with 3 repetitions of each combination, obtaining a total of 90 experimental tests, 45 for each experimental response. These averages are shown in Table 4.

### 3.1. Flexural Strength Analysis 

The aim of the analysis is based on variance analysis and consists of finding a treatment different from the rest for this experimental response, which will be best for this property.

For the variance analysis, the following hypotheses are posed

- H_0_: The population means of flexural strength is statistically equal.
- H_1_: At least one of the means of flexural strength is statistically different.

Table 5 determined that the significance of the model is equal to 0.000 and less than the predefined significance of 0.05, affirming that the null hypothesis is rejected and that there is an optimal case.

**Figure 1:**
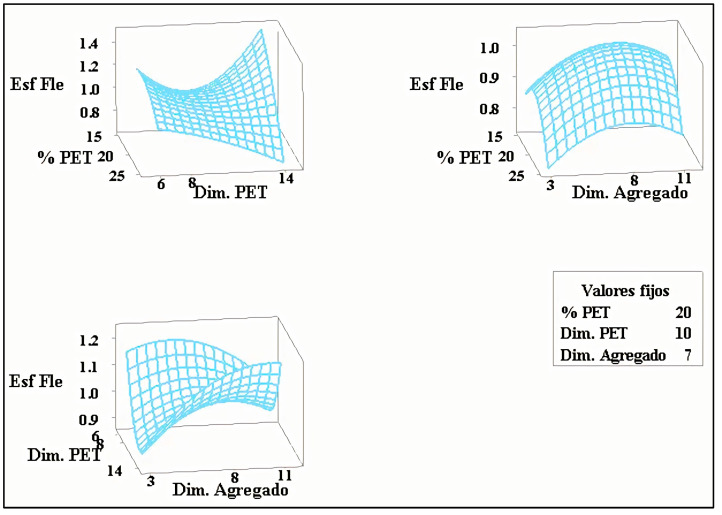
The surface of flexural strength response.

Figure 1 shows that the highest points of the flexural strength can be visually estimated. However, for a more precise estimate, the desirability function is invested.

### 3.2. Compression Strength Analysis

Optimization requires finding a particularly different treatment from the rest for this experimental response, cataloging as the best. The hypothesis for this end is posed as follows:

- H_0_: The population means of the compression strength are statistically equal
- H_1_: At least one of the population means of compression strength is statistically different.

In Table 6, the value of the calculated significance of the model is reviewed at 0.001 and less than the predefined significance of 0.05, leading to the null hypothesis being rejected, ensuring that there is a different treatment from the rest, and letting us state that there is an optimal case.

**Figure 2:**
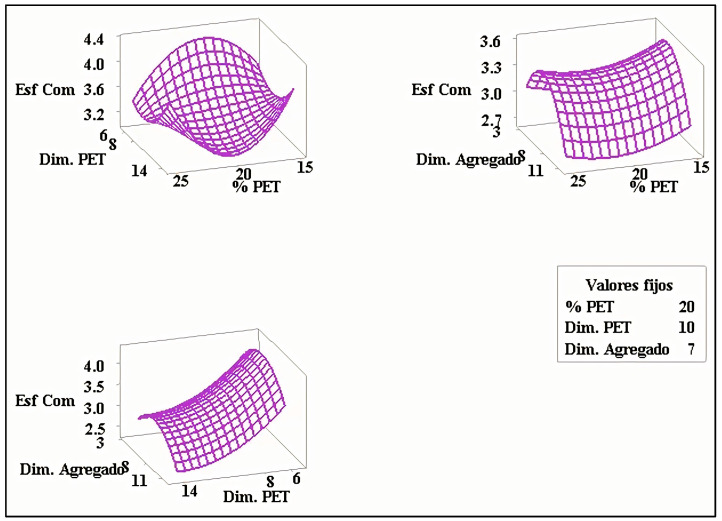
Compression strength response surface.

In Figure 2, the highest compression strength points can be visually estimated; however, for a more precise estimate, the desirability function is used.

### 3.3. Multiple Optimization

Once the individual optimum points have been achieved, wherein the individual desirability values of the experimentally analyzed responses are included, the overall desirability is carried out.

**Figure 3:**
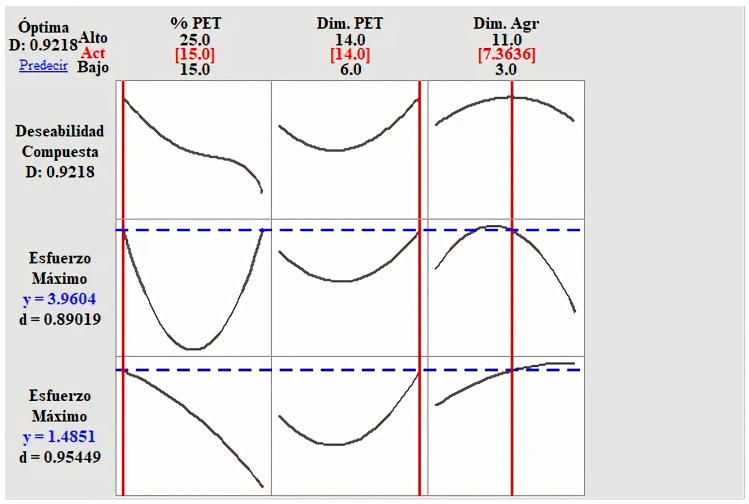
Overall desirability of the flexural and compression strength.

Overall desirability considers all possible combinations within the experimental region described by the levels of the three factors analyzed. Figure 3 shows the lines of each desirability and the overall desirability, where the optimal values of each factor are specified.

The global optimum point is presented with the dosage of 15% PET in the mixture with a dimension of 14 mm and the addition of a dimension of 7.36 mm. All this lets us obtain a manufactured masonry veneer with the best mechanical properties, as shown in Table 7.

The results obtained after optimization are presented and reviewed with respect to the results of the tests on the control specimens, which are commercial facades. The reference values were obtained from eight specimens. The information is presented in Table 8.

### 3.4. Scanning Electron Microscopy (SEM) Analysis

Based on the global optimization obtained, where the ideal dosage of factors that make up the masonry veneers material is estimated (Table 6, Factors) and those that generate the best mechanical properties in flexion and compression (Table 6, Results), it is important to analyze the morphology of the material, as well as the interface (matrix vs. reinforcements) between the different factors or components that make up the composite material.

For this, an SEM analysis was carried out from samples of the cross-section of the material (Table 2—Cases 10 and 5) which are, respectively, those with the greatest and least proximity to the ideal results obtained in the global optimization (Table 9).

**Figure 4:**
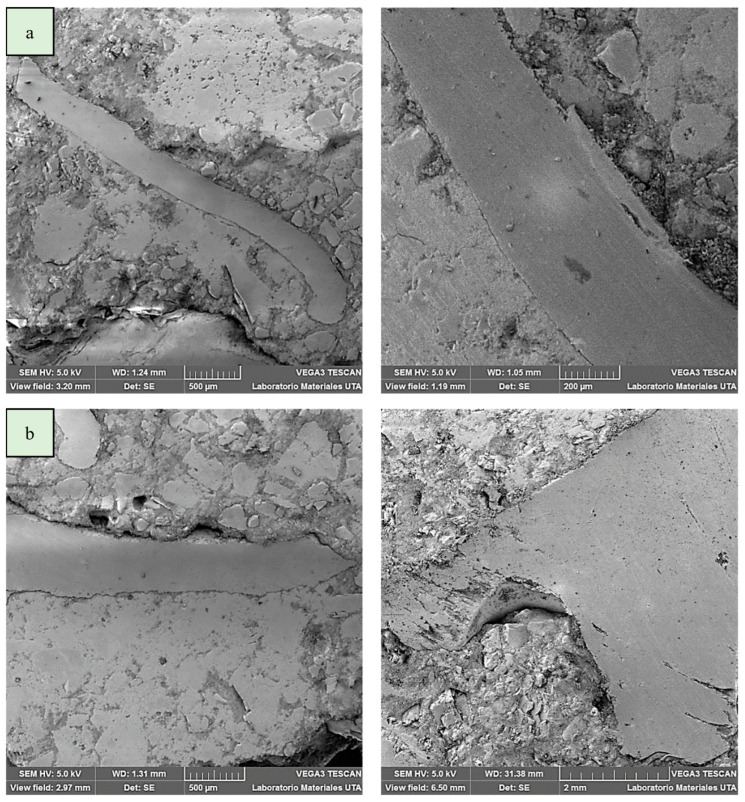
SEM morphology of the cross-section of reference materials of the investigated masonry veneers: (**a**) shows the excellent adhesion and cohesion characteristics between the PET fibers and the matrix (Table 7, Case 10 ≈ OG); (**b**) shows a medium adhesion and cohesion between the PET fibers and the matrix (Table 7, Case 5 ≠ OG).

From the SEM analysis it can be seen in Figure 4a excellent adhesion and cohesion characteristics between the PET fibers and the matrix (Case 10); both factors are important for load absorption and, with it, the improvement of mechanical performance, while Figure 4b shows a medium adhesion (Case 5), which causes a faster failure under the action of bending and/or compression loads.

## 4. Discussion

Hassan, Fattah and Tamimi [17,21] focused on the use of DOE, specifically response surface methodology (RSM), to evaluate the effect of the dispersion protocol on cement-based composite materials’ characteristics. They obtained a coefficient of determination of 82.67%, which was adequate to have valid and comparable values of the combinations made in the article in question. For our response surface model, the coefficient of determination was 90.08%, which better explains the variability of the flexural strength. In addition, the determination coefficient with the regression model generated by the response surface methodology also explains 67.15% of compression strength variability.

After applying the models to each of the properties, the responses were optimized. Kumar [24,28] applied a multi-objective optimization technique in an efficient manner, letting him find the cost-effective and energy-efficient CFC mixture. With this optimization technique and through the models generated with ANOVA, it was found that the optimal point in the dosage is to use 15% PET in the mixture, a PET size of 14 mm and an aggregate size of 10.5152 mm. According to the model, the maximum flexural strength would be 1.5361 MPa. This value is very close to the maximum strength obtained experimentally when testing masonry veneers made of reinforced concrete with 25% PET replacing the aggregates, with a size of 14 mm and an aggregate of 8 mm, obtaining a flexural strength of 1.5995 MPa. The flexural strength of the fabricated masonry veneers with 25% PET composite material, 14 mm PET and 8 mm aggregate is also 56% higher than the bending strength of the commercially available fabricated masonry veneers with 0.70 MPa. The increase is 0.89 MPa when testing the manufactured masonry veneer.

With the same procedure described above, it is known that the most optimal point in the dosage is when using 19.34% PET in the mixture with a dimension of 6 mm and an aggregate of 6.31 mm to obtain a wall with a compression strength of 4.32 MPa. This is very close to the compression strength obtained experimentally with masonry veneers manufactured using 20% PET substitution in the mixture with a dimension of 6 mm and an aggregate of 11 mm, obtaining a compression strength of 4.31 MPa.

The compression strength of the masonry veneer manufactured with 20% PET with a dimension of 6 mm and an aggregate of 11 mm increased the compression strength of the commercially manufactured masonry veneer by 52.62% with 2.04 MPa. The increase presented is 2.26 MPa when testing the fabricated masonry veneers.

The masonry veneer designed with the optimum combination has a flexural strength of 1.48 MPa and a compression strength of 3.9 MPa. The following are presented tests of commercial masonry veneer, which have similar characteristics and dimensions to those developed in this research; these masonry veneers have a flexural strength of 0.70 MPa and compression stress of 2.04 MPa, understanding the benefits of incorporating recycled PET in the manufacture.

Recycled PET was used due to the ease and magnitude of plastic bottle waste; however, reviewing the feasibility of using another type of polymer as an aggregate, we have the work of Abu-Saleem [33], which investigates the use of PET, HDPE and PP, concluding that, for compression strength and flexural strength, the addition of PET10%, PP10% and PET20%, increases the tensile strength of the material, HDPE and PP, concluding that, the addition of PET10%, PP10% and PET20% increases the tensile strength by 22.4%, 9.2% and 6.6%, respectively, with respect to the control mix.

Furthermore, although it was not a subject of review for the present research, the origin of the plastic material added to any ceramic matrix can be relevant since virgin polymeric materials improve the performance of materials. Although on the other hand, the use of recycled material, if it provides benefits in the economic issue and certain properties [34], may present drawbacks in the compatibility between the ceramic matrix and the plastic material, Al-Mansour et al. [35] explain that this can be improved by incorporating the additive ethylene vinyl acetate (EVA) to the plastic as a coating.

Even though the stability and durability of the masonry veneers manufactured for this work were not investigated, it is worth mentioning that given the structure and properties of PET, the addition of its particles to concrete would result in higher durability. This is because PET is chemically inert and very stable in alkaline media such as concrete [36]. Furthermore, adding recycled plastic particles to concrete reduces its permeability, which also increases its durability compared to the samples that only include natural aggregates [37]. Despite this, as previous authors have suggested, the durability of the materials such as the ones studied here should be further investigated.

## 5. Conclusions

The present experimental study performs optimization by response surface methodology to determine the optimal dosage of input factors to produce manufactured masonry veneer and perform the flexural strength and compression strength tests by mixing river sand, water, aggregate, Portland cement and crushed recycled PET, varying its percentage of addition in the mixture as well as its dimension and the dimension of the aggregate. We can draw the following conclusions from the results obtained in this investigation.

The regression model by response surface methodology explains the variability of the flexural strength and compression strength by 90.08% and 67.15%. The hypothesis also contrasts with a calculated significance close to 0% being achieved. This value is very appreciably below 5%.

The regression model with up to second-degree terms and up to third-degree interactions allows sources of variability to be involved, which permits the error to be displaced due to lack-of-fit. This makes the most of the information obtained with the trials efficiently, evidencing statistically enriching information.

The desirability function is an objective method to efficiently take advantage of a combination. It analyses flexural and compression strength, involving all the points within the experimental region delimited by the input factor levels and optimizing the number of specimens. Composite material dosage is, thus, the most significant factor for all the mechanical properties considered.

The global optimum optimization point is presented when manufactured masonry veneers are manufactured with a dosage of 15% PET in the mixture with a dimension of 14 mm and aggregate of 7.36 mm. With all this, it is possible to better use the walls’ mechanical properties with a flexural strength of 1.48 MPa and a compression strength of 3.9 MPa.

## 6. Contribution and Applications

With the development of the research, it is possible to manufacture a composite material consisting of concrete plus the addition of recycled crushed polyethylene terephthalate obtained from plastic drink bottles, in addition to having a structured procedure based on the recommendations of the Institute of Occupational Safety and Hygiene (INSHT).

In practice, it is proposed to give utility to the PET obtained by recycling plastic bottles, minimizing environmental pollution and giving it a new use. The composite material will also be directed toward the construction industry, with a focus on masonry veneers.

By optimizing the composite, a 10.85% lighter material is achieved, with up to 56% better properties (flexion) and better surface finish, compared to commercial masonry veneers.

## Figures and Tables

**Table 1:** Characteristics of the concrete and crushed recycled PET used in the manufactured composite masonry veneers.

Material	Dimension (mm)	Characteristic	Density (g/cm^3^)	City of Origin
Aggregate 1	3	Granule	2.122	Latacunga
Aggregate 2	8	Granule	1.315	Latacunga
Aggregate 3	11	Granule	1.146	Latacunga
PET 1	6	Crushed	1.341	Santo Domingo
PET 2	8	Crushed	1.341	Santo Domingo
PET 3	14	Crushed	1.341	Santo Domingo
River sand	3	Granule	2.697	Santo Domingo
Holcim Cement	---	Powder	2.986	Ambato
Artesil 1 (Silicone Elastomer)	---	Liquid	1.20	Cuenca
Silicone Emulsion	---	Liquid	1	Cuenca

**Table 2:** Combinations of materials used to obtain the manufactured masonry veneers made of concrete, aggregates and crushed recycled PET.

Combination	River Sand (%)	Crushed Recycled PET		Aggregate Dimension (mm)
		Replacement (%)	Dimension (mm)	
1	80	20	6	3
2	85	15	8	3
3	75	25	8	3
4	80	20	14	3
5	85	15	6	8
6	75	25	6	8
7	80	20	8	8
8	80	20	8	8
9	80	20	8	8
10	85	15	14	8
11	75	25	14	8
12	80	20	6	11
13	85	15	8	11
14	75	25	8	11
15	80	20	14	11

**Table 3:** Factors and responses of the Experimental Design—Box–Behnken.

Factors	PET (%)	15
		20
		25
	PET dimensions (mm)	6
		8
		14
	Aggregates dimensions (mm)	3
		8
		11
Responses	Flexural tests (MPa)	
	Compression Test (MPa)	

**Table 4:** Flexural strength and compression test results.

Combination	Crushed Recycled PET		Dry Thickness (mm) (Aggregate)	Flexural Strength (Mpa)	Compression Strength (Mpa)
	Replacement (%)	Dimension (mm)			
1	20	6	3	1.1748	3.9730
2	15	8	3	0.8592	3.3390
3	25	8	3	0.7375	3.1200
4	20	14	3	0.9167	2.5344
5	15	6	8	1.1656	3.9322
6	25	6	8	0.9770	3.3158
7	20	8	8	1.0501	2.9262
8	20	8	8	0.9310	4.0748
9	20	8	8	1.0961	3.0119
10	15	14	8	1.4881	4.1023
11	25	14	8	0.6686	3.8727
12	20	6	11	1.0727	3.6035
13	15	8	11	0.9257	2.5564
14	25	8	11	0.8261	2.9041
15	20	14	11	1.2201	2.5107

**Table 5:** Analysis of flexural strength variance.

Source of Variability	D.F	Sum of Squares	Mean Square	F-Value	*p*-Value
Model	9	0.6480	0.0720	20.18	0.000
% PET	1	0.0134	0.0134	3.76	0.067
Dim. PET	1	0.0012	0.0012	0.36	0.556
Dim. Aggregate	1	0.0174	0.0174	4.88	0.039
(% PET) · (% PET)	1	0.0446	0.0446	12.51	0.002
(Dim. PET) · (Dim. PET)	1	0.0960	0.0960	26.94	0.000
(Dim. Agr.) · (Dim. Agr.)	1	0.0283	0.0283	7.96	0.011
(% PET) · (Dim. PET)	1	0.1091	0.1091	30.60	0.000
(Dim. PET) · (Dim. Aggregate)	1	0.0451	0.0451	12.65	0.002
(% PET) · (Dim. PET) · (Dim. PET)	1	0.0849	0.0849	23.81	0.000
Lack-of-fit	3	0.0025	0.0008	0.21	0.887
Pure error	17	0.0687	0.0040	-	-
TOTAL	29	0.7194	-	-	-

**Table 6:** Analysis of compression strength variance.

Source of Variability	D.F	Sum of Squares	Mean Square	F-Value	*p*-Value
Model	8	7.1873	0.8984	5.37	0.001
% PET	1	0.1286	0.1286	0.77	0.391
Dim. PET	1	3.2038	3.2038	19.14	0.000
Dim. Aggregate	1	0.4842	0.4842	2.89	0.104
(% PET) · (% PET)	1	0.1580	0.1580	0.94	0.342
(Dim. PET) · (Dim. PET)	1	0.7647	0.7647	4.57	0.045
(Dim. Agr.) · (Dim. Agr.)	1	1.8762	1.8761	11.21	0.003
(% PET) · (Dim. PET)	1	0.0748	0.0748	0.45	0.511
(% PET) · (% PET) · (Dim. PET)	1	2.6541	2.6541	15.85	0.001
Lack of fit	4	0.5495	0.1373	0.79	0.549
Pure error	17	2.9663	0.1745	-	
TOTAL	29	10.7031	-	-	

**Table 7:** Optimization point Global Optimum (GO).

	Variable	Value	Unit
Factors	PET percentage	15	%
	PET dimension	14	mm
	Aggregate dimension	7.36	mm
Results	Flexural Strength	1.48	MPa
	Compression Strength	3.96	MPa

**Table 8:** Analysis of physical—mechanical behavior of the masonry veneers: commercial vs. ecological (PET).

**Commercial masonry veneer** **Density = 2.7 g/m^3^** **(8 specimens tests)**	** 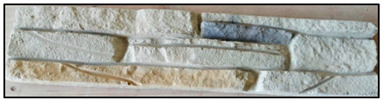 **		
**Ecological (PET) masonry veneer** **Density = 1.8 g/m^3^** **(90 specimens tests)**	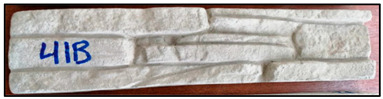		
**Compression test** **NTE INEN 1573** 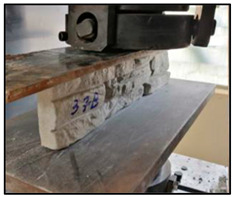	Compression Strength (Shimadzu Machine Concrete 2000x)	Commercial	2.04 MPa
		Ecological (PET)	3.96 MPa
	Failure mode: Common/non-explosive. Properties: Excellent toughness.	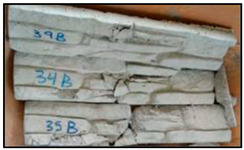	
**Flexural test** **NTE INEN 2554** 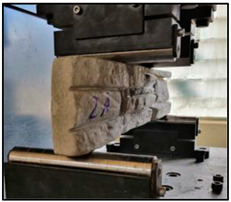	Flexural (four point) Strength (Shimadzu Machine Concrete 2000x)	Commercial	0.70 MPa
		Ecological (PET)	1.48 Mpa
	Failure mode: Mode I—Typical by flexion. Properties: High ductility/higher toughness.	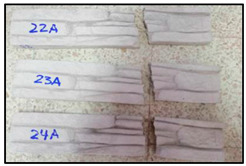	

**Table 9:** Obtaining specimens for SEM analysis.

	Variables	Global Optimum (GO) Table 6	Referential Case for SEM ≈ GO Table 2—Case 10	Referential Case for SEM ≠ GO Table 2—Case 5
Factors	PET (%)	15	15	15
	PET Dim. (mm)	14	14	6
	Aggregate Dim. (mm)	7.36	8	8
Results	Flexural Strength (MPa)	1.48	1.38	1.10
	Compression Strength (MPa)	3.96	3.96	3.45

## Data Availability

The data presented in this study are available on request from the corresponding author.
